# *Drosophila* as a Model System for Studying of the Evolution and Functional Specialization of the Y Chromosome

**DOI:** 10.3390/ijms23084184

**Published:** 2022-04-10

**Authors:** Alexei A. Kotov, Sergei S. Bazylev, Vladimir E. Adashev, Aleksei S. Shatskikh, Ludmila V. Olenina

**Affiliations:** Institute of Molecular Genetics of National Research Center «Kurchatov Institute», 123182 Moscow, Russia; kotov_alexei@mail.ru (A.A.K.); bazylevser@gmail.com (S.S.B.); adashev.vladimir@gmail.com (V.E.A.); shackih@yandex.ru (A.S.S.)

**Keywords:** *Drosophila*, Y chromosome, piRNA pathway, rDNA, intron gigantism, azoospermia, transposable elements

## Abstract

The Y chromosome is one of the sex chromosomes found in males of animals of different taxa, including insects and mammals. Among all chromosomes, the Y chromosome is characterized by a unique chromatin landscape undergoing dynamic evolutionary change. Being entirely heterochromatic, the Y chromosome as a rule preserves few functional genes, but is enriched in tandem repeats and transposons. Due to difficulties in the assembly of the highly repetitive Y chromosome sequence, deep analyses of Y chromosome evolution, structure, and functions are limited to a few species, one of them being *Drosophila melanogaster*. Despite Y chromosomes exhibiting high structural divergence between even closely related species, Y-linked genes have evolved convergently and are mainly associated with spermatogenesis-related activities. This indicates that male-specific selection is a dominant force shaping evolution of Y chromosomes across species. This review presents our analysis of current knowledge concerning Y chromosome functions, focusing on recent findings in *Drosophila*. Here we dissect the experimental and bioinformatics data about the Y chromosome accumulated to date in *Drosophila* species, providing comparative analysis with mammals, and discussing the relevance of our analysis to a wide range of eukaryotic organisms, including humans.

## 1. Introduction

**Figure 1:**
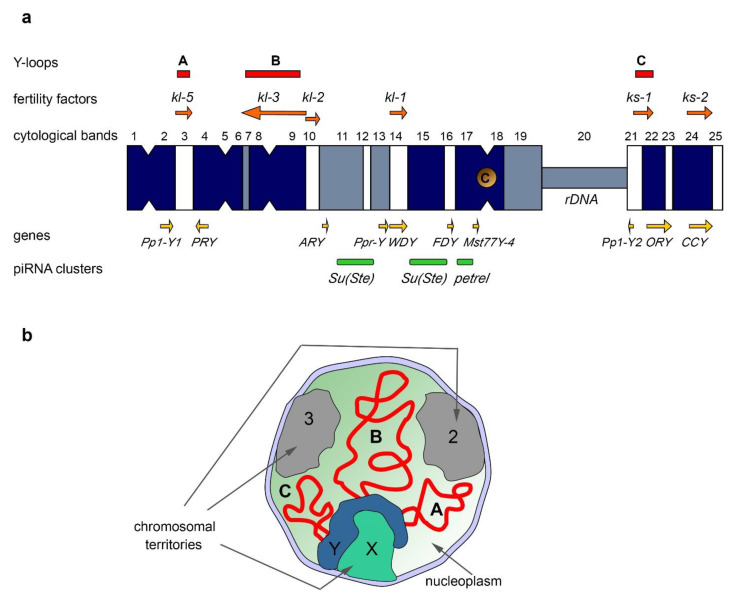
(**a**) The scheme of the Y chromosome of *D. melanogaster*. Cytological bands are shown in accordance with Hoechst 3325B banding, white bands represent no fluorescence. From top to bottom: cytological location of fertility factor genes (orange arrows) and corresponding Y-loops (red bars); Y chromosome scheme with cytological bands indicated; cytological location of the centromere (C), *rDNA* loci, and several genes (yellow arrows); cytological location of largest piRNA clusters (green bars). The scheme is modified from [6] according to data from [2,4,7]. (**b**) The scheme of Y-loop distribution in the nucleus of a primary spermatocyte in G2 phase. Chromatin forms three discrete chromosome territories that are adjacent to the inner nuclear membrane and consisted of paired chromosomes 2 and 3, and also X/Y. Transcriptionally active Y-loops A, B, and C are distributed in the nucleoplasm (thick red lines).

Compared to the autosomes and the X chromosome, the Y represents a unique chromatin landscape undergoing dynamic evolutionary change. The Y chromosomes of different animals almost entirely consist of heterochromatin and, as a rule, contain few functional genes, but are enriched in simple repeats and transposons. To date the uncovering of their evolutionary history and studying of their multiple biological manifestations using model organisms allow us to better understand the specific functions of Y chromosomes in animals. However, deep analysis of Y chromosomes is restricted to a few species, one of them being *Drosophila melanogaster*. The Y chromosome of *D. melanogaster* comprising about 40 Mb of heterochromatic DNA contains about 14 protein-coding genes (Figure 1a) mainly acquired from the autosomes, whereas 80% of its sequence is represented by tandem repeats [1,2,3,4]. A recently developed approach using single-molecule long-read sequencing allows researchers to build a new genome assembly and to study *D. melanogaster* Y composition in detail [4]. Many functional Y-linked genes are found to be duplicated and most of these duplicated copies are pseudogenes not supported by natural selection. The short right arm of the Y chromosome is filled by multiple rDNA loci and their intergenic spacer (IGS) repeats. Satellites, or simple tandem repeats, presumably constitute about 65% of the entire Y chromosome, and Long Terminal Repeats (LTR) and Long Interspersed Nuclear Element (LINE) transposable elements comprise 18% and 7% of the total Y sequence, respectively. The Y chromosome has a 1.4–1.8-fold enrichment in retrotransposon content compared to 10% of LTRs and 5% of LINEs in the rest of the genome [4]. Whereas Y chromosomes exhibit high structural divergence between even closely related species [5], shared developmental trajectories provide convergent evolution of Y chromosomes across different organisms.

This review attempts to cover some significant aspects of Y chromosome functioning that have been highlighted in the last decades owing to investigations in *Drosophila*. Here we survey comparative evolutionary history of the fly and human Y chromosomes, peculiarities of transcription of giant genes, such as genes, encoding fertility factors in *Drosophila*, differential expression of sex-linked rDNA loci, and functions of Y-linked piRNA clusters ensuring sex-specific piRNA silencing. Our comparative analysis will provide further insight into the properties of the Y chromosomes in both insects and mammals.

## 2. Comparative Evolutionary History of the Fly and Human Y Chromosomes

### 2.1. Y Chromosome Differentiation and Functions in Flies

**Figure 2:**
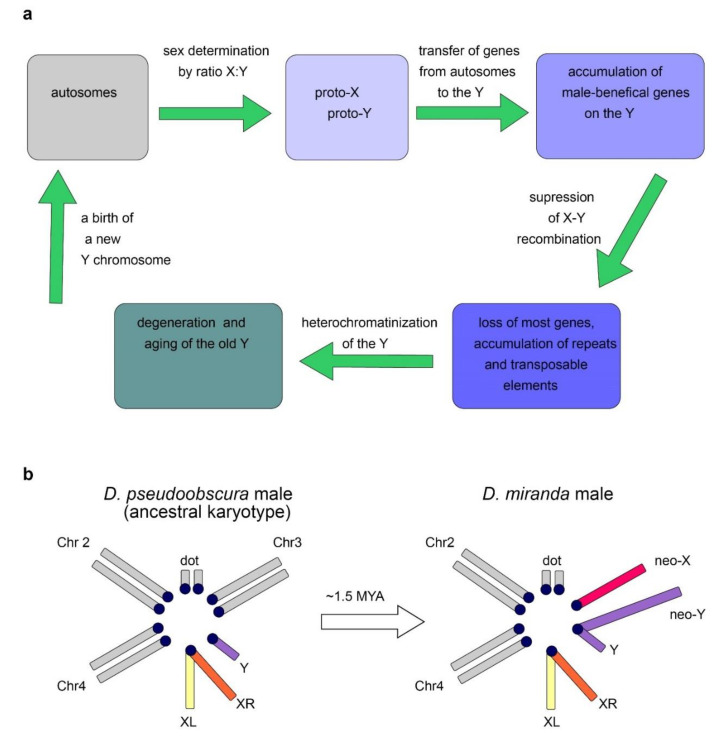
(**a**) Putative Y chromosome life cycle in genus *Drosophila*. It starts with the origin of proto-Y from the autosome, then goes throughout accumulation of genes necessary for spermatogenesis and male fitness, cessation of the X-Y recombination, degeneration of the bulk of acquired genes, heterochromatinization, aging, and arising from autosomes repeatedly. (**b**) The scheme of the karyotype of *D. miranda* males and its close relative species *D. pseudoobscura*, from which *D. miranda* diverged about 2 MYA. In *D. miranda*, the fusion of chromosome 3 with the ancestral Y chromosome created the neo-Y chromosome about 1.5 MYA. XL and XR indicate the left and right arm of the ancestral X chromosome. The scheme is modified from [14].

The Y chromosome is a sex chromosome found in males of different groups of animals, including mammals and Diptera. Whereas in mammals the development of an organism according to the male type is determined by the presence of a functional Y chromosome, in *Drosophila*, sex is determined by the ratio of the number of X chromosomes to the number of the autosomes: normally, the presence of two X chromosomes triggers development according to the female type, and one—according to the male type [8,9]. Thus, individuals with the XXY genotype are female in flies and male in mammals, while X0 are female in mammals and male in flies. In *Drosophila* with the X0 karyotype, there are no severe structural or functional body disorders, except for male sterility [6]. Fly Y chromosome is not involved in sex determination. Several genes functioning in sex determination of flies, such as *Sex-lethal (Sxl)*, *transformer (tra)*, *transformer-2 (tra2)*, and others, have been found to date. Sex-specific mRNA splicing of major *Drosophila* sex-determining genes is a complex process providing female-specific transcripts triggering female-type development [8,9]. In Diptera, the Y chromosomes arose from the autosomes repeatedly (Figure 2a), which provides good material for studying parallel processes of convergent evolutionary development [10,11,12]. While the gene content of the autosomes in Diptera is conserved and originates from a common ancestor, gene composition of the Y chromosomes varies significantly, and the rate of acquisition of new genes often significantly exceeds the rate of their loss [13]. In Diptera, the transfer of genes from other chromosomes contributes to the evolution of the Y chromosome. Relatively young, newly formed Y chromosomes of flies, being formed from tens of thousands of years to a few million years ago, maintain the structure and genes of the ancestral autosome, while most of the genes on the old fly Y chromosomes have been acquired subsequently due to a transfer from the autosomes or the X chromosome. Old Y chromosomes such as in *D. melanogaster* that presumably have been persisted for long time (several decades of millions of years) are often highly heterochromatic, contain a large amount of repetitive DNA, and their genes undergo degeneration [10,12].

*Drosophila miranda* contains a pair of young neo-sex chromosomes that were born ~1.5 million years ago (MYA) after splitting from the closely related species *Drosophila pseudoobscura*. *D. miranda* is an excellent model for studying early sex chromosome differentiation given the appearance of new advanced methods of genomic sequencing and assembly. Its neo-sex chromosomes have been created by the fusion of the former autosome 3 with the ancestral, degenerated Y chromosome of this clade (Figure 2b) [14]. Initial stages of Y evolution are characterized by massive amplification of distinct classes of genes. The neo-Y chromosome of *D. miranda* initially contained about 3000 protein-coding genes, but during its evolution, it has acquired over 3200 genes, primarily by tandem amplification of protein-coding genes that were ancestrally present on the chromosome. Testis-specific and dosage-sensitive genes appear to have amplified and have been fixed on the neo-Y to facilitate male fitness. The neo-X and neo-Y chromosomes in *D. miranda* still maintain a high homology across their length, with up to ~98% sequence identity in homologous regions [14,15]. A cohort of meiosis-related multi-copy Y-linked genes have independently co-amplified on the X, and their expansion is probably driven by conflicts over segregation. These co-amplified X-Y genes are highly expressed in spermatogenesis and are significantly enriched for meiosis-related and RNA interference functions. Drive systems on the sex chromosomes presumably include distorter elements causing meiotic drive and biasing progeny sex ratio, and suppressor elements evolving on the opposite chromosome for preventing meiotic drive [16]. It has been proposed that their co-amplification is driven by X/Y antagonism providing increased transmission of a certain sex chromosome to the next generation, where suppression of chromosome can be mediated by sequence homology between the suppressor and distorter elements with participation of the RNA interference pathways. It has been suggested that newly emerged sex chromosomes are a battleground for meiotic drive and X-Y inter-chromosomal conflicts [14,16].

In some *Drosophila* species, such as *D. pseudoobscura*, the old Y chromosome has become part of the autosome, while the neo-Y has been presumably formed de novo several MYA (Figure 2a) [12,17]. At the same time, the gene content of the Y chromosomes in *D. pseudoobscura* and *D. melanogaster* is completely different, except that both species maintain various Y-linked genes necessary for male fertility. The same pattern can be seen upon comparison of other Diptera species: despite the different evolutionary history, the functions of most Y-chromosomal genes are related to the male reproductive system [5,12]. A recent report about *D. willistoni* confirms the previously observed Y duplication of autosomal or X-linked testis-specific genes and the prominent role of gene gains in the evolution of *Drosophila* Y chromosomes [18].

### 2.2. Origin of the Y Chromosome in Mammals and Sex Determination

In many animals, sex is determined by a pair of heteromorphic X and Y chromosomes. According to modern concepts, sex chromosomes originate from an ancestral pair of autosomes, one of which acquires a sex-specific gene, which starts the process of differentiation of the sex chromosomes. In mammals, this event occurred only once in the common ancestor of marsupials and placentals prior to their splitting, about 160–180 MYA [19,20,21,22]. The proto-Y chromosome of all mammals (from kangaroo to human) arose from a single autosome in which one of the alleles of the *SOX3* gene, as a result of a mutation, became the sex-determining gene *SRY* (*Sex Reversal Y*) [23,24]. It is important to note that this gene is not responsible for all sex characteristics alone. The product of the sex determination gene only provides a switch, triggering a certain pathway of development. Unique evolutionary forces facilitated the selection and accumulation of male-beneficial mutations around the *SRY* locus, and the linkage between them was supported by selective pressure to avoid crossing over between the proto-Y and proto-X [25]. As a rule, if the dominant allele causes the development of a male, then the chromosome in which it is located becomes the Y chromosome (and its homolog is called the X). In birds, males are the homogametic sex (ZZ) and females are the heterogametic (ZW) [21]. Currently two main hypotheses about sex determination in birds are presented. One of them postulates the presence of the key gene controlling ovarian development or inhibiting testis differentiation in the W chromosome, while the other one proposes the number of Z chromosomes as a key sex-determining factor [21,26]. In last case, sex determination is thought to be provided by a sex chromosome gene dosage mechanism, and the most likely sex determinant is the Z chromosomal gene *DMRT1* encoding transcription factor. Recently it was shown that male chicken (ZZ) with a single functional copy of *DMRT1* (other was deleted by a CRISPR-Cas9-based monoallelic targeting approach) developed ovaries in place of testes. It indicated that DMRT1 is the key sex determination switch in birds essential for testis development [26]. In addition, it was found that the synthesis of estrogen is also an essential factor in primary sex determination in chicken, and that estrogen production is controlled by expression of *DMRT1* [26]. These data support the second hypothesis that the dosage of genes on the Z chromosome determines the sexual differentiation in birds [26].

### 2.3. Evolutionary Factors and Forces Determining the Structure and Functional Specialization of the Y Chromosome

The loss of recombination leads to the inefficiency of natural selection and causes the ensuing accumulation of Y-linked loss-of-function mutations, chromosome-wide gene decay, and amplification of repetitive DNAs [27,28,29,30]. In parallel to the loss of genes, Y chromosomes have accumulated large amounts of DNA repeats, and the *D. melanogaster* old Y chromosome mainly consists of heterochromatin (Figure 2a) [4,21]. Despite the human Y chromosome having undergone a rapid decay early in evolution, its massive degeneration then dramatically stopped. Genes that remained intact currently show remarkable stability, and no human Y-linked genes have been lost during the last 44 million years [22,31]. The maintenance of human Y-linked genes is mainly associated with two functional categories: genes essential for male reproductive functions and dosage-sensitive ubiquitous housekeepers [32]. Studies of males with Y deletions have allowed researchers to identify three ‘azoospermia factor’ (*AZF*) regions, *AZFa*, *AZFb*, and *AZFc*, and partially map within them the genes essential for spermatogenesis [33]. The *AZFa* deletions affecting the *DBY* gene cause the most severe azoospermia phenotype, exhibiting a complete loss of testis germline cells accompanied by the maintenance of somatic Sertoli cells (the so-called Sertoli Cell-Only Syndrome; SCOS) [34,35,36].

As in fruit flies, mammalian Y chromosomes also exhibit gene amplification, with the amplicon structures predominantly containing genes with testis-specific functions [37]. The structure of such genes is maintained by intra-chromosomal gene conversion. The amplicon region of the human Y chromosome contains eight massive palindromes ranging in length from 9 kb to 1.45 Mb with nucleotide identity of the arms over 99.9% [38,39,40]. Due to the presence of repeating structures, local intra-chromosomal gene conversion is possible, as well as intra- and inter-chromatid exchange. These mechanisms partially compensate for the lack of recombination with the X chromosome by eliminating harmful mutations. At the same time, inter-chromatid recombination can in some cases lead to the formation of isodicentric chromosomes formed by homologous crossing over between opposing arms of palindromes on sister chromatids. This may be accompanied by the loss of certain regions containing genes essential for spermatogenesis, and in some cases can lead to the loss of the Y chromosome during cell division with clinical consequences ranging from spermatogenic failure to sex reversal and Turner syndrome [41].

The loss of the ability to recombine plays a key role in establishing the structure of the Y chromosome, because recombination could lead to a disruption of sex determination and the formation of infertile intermediate variants [30,42]. Conversely, mutations that prevent recombination between proto-X and proto-Y, such as inversions, deletions, or accumulation of repeats, are supported by selection. Reducing the ability of recombination with the homologous X chromosome dramatically accelerated the evolution of the Y chromosome preventing the elimination of emerging mutations via crossing over, while the X chromosome has retained the ability to cross over in the homogametic sex. This led to the degeneration of most of the original Y-chromosomal genes, and multiple deletions caused a significant size decrease with a relative increase in the proportion of non-coding heterochromatic regions. The rapid evolutionary degeneration of the Y chromosomes, typical in a wide range of species, leads to the hypothesis that in the future the human Y chromosome may disappear altogether. This hypothesis is based not only on extrapolation, but is also indirectly supported by precedents in the evolution of some species including multiple fishes, reptiles, grasshoppers, cockroaches, and dragonflies [43,44,45]. However, other researchers claim that human Y degeneration stopped millions of years ago and currently nothing threatens Y chromosome survival [46].

### 2.4. Dosage Compensation System Contributes to Y-Linked Gene Maintenance

As a rule, a single gene copy appears to be enough to provide development and life-cycle maintenance of diploid animals; however, a small cohort of genes exhibits a high sensitivity in case of decreased gene dosage. This phenomenon is known as haploinsufficiency, and it is associated with many developmental disorders in human [47,48,49]. Comparing the evolution of flies and humans, one could assume that the Y-chromosomal genes, which have homologues on the X chromosome and do not directly contribute to the functioning of the male reproductive system, are relics that will disappear over time, as has apparently happened in flies. However, their maintenance can be determined by the peculiarities of the dosage compensation system in mammals. In male flies, the genes of the only X chromosome are overactivated in somatic tissues, eliminating the problem of haploinsufficiency and potentially lethal imbalance between the X and autosome transcriptional level in the two sexes. Therefore, in flies, X activation may eventually compensate for haploinsufficient homologous genes lost on Y, which is impossible in mammals. In contrast, in female mammals, inactivation of one of the two X chromosomes occurs. However, according to various estimates and in distinct types of human cells, 20–30% of genes of inactive X chromosome escape the inactivation [50,51]. In mammals, haploinsufficient Y-chromosomal genes have X-chromosomal homologues that avoid inactivation during dosage compensation in females, which indicates the need for their expression on both sex chromosomes to ensure normal functions in the body. Thus, in males, these dosage-sensitive genes cannot disappear from the Y chromosome without negative consequences, and they can survive under selective pressure [31]. This hypothesis is supported by the maintenance of functional X-Y gene pairs associated with housekeeping regulatory functions such as lysine demethylation, stem cell self-renewal, splicing, translation initiation, and deubiquitylation [31,32,50,52]. Strict dosage requirements for sex-linked genes are demonstrated in the case of Turner syndrome (exhibiting X0 karyotype or mosaicism) and Klinefelter syndrome (XXY), since such genes have been haploinsufficient or overexpressed, respectively, in these karyotypes [51]. Turner syndrome is a genetic condition caused by complete or partial loss of the second sex chromosome in human. Half the patients with Turner syndrome have the X0 karyotype (monosomy of the X chromosome), the other half exhibits mosaicism or a presence of the fragmented X or Y chromosomal material and other more complex karyotypes [53,54]. Studies of manifestations of this syndrome indicate that the functions of the Y chromosome consist not only of ensuring the normal functioning of the male reproductive system. Due to the absence of the *SRY* gene, which is the key to triggering male-type development, patients with this syndrome are exclusively female, with multiple body disorders: small size, rudimentary ovaries and infertility, pathologies of the cardiovascular system, autoimmune disorders, increased risk of developing diabetes, and cognitive impairment [54]. Individuals with Klinefelter syndrome are infertile as a result of excess gene dosage of X escape genes, and abnormal meiotic pairing of the sex chromosomes. An atypical number of X or Y chromosomes (XXY, XXX, or X) contributes to spatial chromosome conformation changes and leads to disruption of DNA methylation patterns of autosomal genes, causing distinct disease phenotypes: mental illness, cancer, and disrupted fertility [51].

### 2.5. Convergent Nature of the Evolution of Y Chromosomes

Despite their independent evolutionary origins in different species, Y chromosomes in species with heterogametic males have a number of similar features: they are usually smaller than X chromosomes, contain significantly fewer genes, most of which are related to the male reproductive system, and also have a relatively large number of repeats and significant areas occupied by heterochromatin. It is worth noting that in species with heterogametic females, such as birds, the sex-specific chromosome W also resembles the Y chromosome in structure, and is characterized by relatively small size, heterochromatinization, and fewer genes [21]. Such common patterns indicate the convergently evolved structural features of these chromosomes. It has been proposed that such convergent evolution is due to the similar nature of the selection pressure. Another common feature—the acquisition of repetitive sequences and the loss of most of the original genes—is associated with accelerated Y evolution due to the loss of recombination with the X chromosome [27]. The difference between the evolution of the Y chromosome in mammals and Diptera is mainly that in Diptera the acquisition of new genes often significantly prevails over the loss of the original ones; although, both processes take place in both groups. Presumably due to slower changes in mammals, the evolutionary processes have not yet reached the point where the Y chromosome has lost all homology with the X chromosome.

Attempts to understand how these patterns are generated can be important not only for fundamental evolutionary biology, but also for biomedical challenges, since Y-chromosomal pathologies in humans differ from other genetic anomalies due to the unique nature of the Y chromosome. It is convenient to study the Y chromosome evolution in species with a rapid generation turnover, as in the *Drosophila* species.

## 3. Fertility Factors and Peculiarities of Giant Gene Transcription

### 3.1. Fertility Factors and Y-Loop Formation in Drosophila

The Y chromosome of *D. melanogaster*, in its current state, contains a few protein-coding genes primarily expressed in the testes. With the aid of classical genetic methods, including X-irradiation, chromosome deficiencies, X-Y translocations, P-element insertion mutagenesis, and complementation assays, it has been discovered that the Y chromosome contains at least six distinct loci required for spermatogenesis and male fertility [55,56,57,58,59,60,61,62,63] (Figure 1a). These six loci encoding the so-called male fertility factors are located both on the long arm (*kl-5*, *kl-3*, *kl-2*, and *kl-1*) and the short arm of the Y chromosome (*ks-1* and *ks-2*) [57,60,64,65]. The *kl-2*, *kl-3*, and *kl-5* genes encode dynein heavy chain proteins that are essential for proper axoneme building in elongating spermatids. Kl-2 is an inner dynein arm heavy chain protein and Kl-3 and Kl-5 are outer dynein arm heavy chain proteins [66,67,68]. Note that *D. melanogaster* has several other dynein heavy chain genes, located on the chromosomes X, 2, and 3 [69]. The axoneme is the microtubule-based main part of the sperm flagellum, a specific motile organelle of spermatids and mature sperm. In the motile flagellum, dynein ATPase motor proteins provide sliding motions between adjacent microtubules, which together produce well-ordered movements [70]. Deficiency of the *kl-3*, *kl-5* or *kl-2* genes leads to loss of the outer dynein structure of the axoneme, and these mutants do not produce motile sperm, resulting in male sterility [65,67,71]. *kl-2*, *kl-3*, and *kl-5* mutant males exhibit clear defects in spermatid morphology and development with the loss of synchronization of their individualization complexes; in addition, they contain short and curled spermatids, nuclei of which are scattered instead of remaining tightly clustered, as in wild-type flies [65]. The gene sequences of *ks-1*, *ks-2*, and *kl-1* had not been identified for a long time, complicating their disruption and studying. However, according to a recent study, the *CCY* gene located near the telomere of the Y short arm is thought to encode male fertility factor *ks-2*. RNAi-knockdown of *CCY* results in short and curled nuclei of elongating spermatids and in male sterility [65,72]. In addition, RNAi-knockdown of the *WDY* gene from the *kl-1* locus leads to male sterility, supporting the conclusion that *WDY* encodes the Kl-1 fertility factor [72].

According to publicly available RNA-seq data, most Y-linked genes begin their expression in third-instar larvae and continue to be expressed throughout the pupal stage with the exception of the *FDY* gene, which is expressed during all developmental stages. Several Y chromosome genes are found to be expressed in imaginal discs, fat bodies, accessory glands, and all of the genes are expressed in the testes [65]. In the *D. melanogaster* testes, generation and transcription of Y-loops take place during primary spermatocyte maturation, coinciding with the data that Y expression peaks in spermatocytes [73]. Transcription of the *kl-2*, *kl-3*, and *kl-5* genes starts in primary spermatocytes and continues during the whole 80–90 h of meiotic G2 phase. Their spliced transcripts are stored in cytoplasmic RNP particles, called kl-granules, in mature spermatocytes along with the ATPase proteins Reptin and Pontin [74,75]. These RNP granules segregate during the meiotic divisions and the stored dynein transcripts undergo delayed translation, occurring post-meiotically. Then the dynein proteins are incorporated into the axoneme during the spermatid elongation process [75].

Fertility factor genes contain unusually large, megabase-sized introns filled with simple satellite repeats in the cases of *kl-5*, *kl-3*, and *ks-1* genes [1,64,76,77,78]. For instance, the *kl-3* gene spans at least 4.3 Mb, while its coding sequence contains only approximately 14 kb [64,76]. The giant introns comprise more than 99% of the whole *kl-3* locus. Transcription from *kl-3*, *kl-5*, and *ks-1* loci in spermatocytes leads to the appearance of lampbrush-like nucleoplasmic structures named Y-loops A (*kl-5*), B (*kl-3*), and C (*ks-1*) (Figure 1a,b), that are visible with the aid of phase-contrast microscopy, and are analogous to those in amphibian oocytes [76]. The *kl-5* locus contains four different satellite repeats; the loop-forming site of *kl-5* accumulates (AAGAG)n, (AAGAC)n, and (AAGAGAG)n repeats, while the *kl-5* non-loop-forming region contains (AATAT)n repeats. Similarly, the *ks-1* locus encompasses (AAGAG)n and (AAGAC)n repeats mapping to the loop-forming region in contrast to (AATAAAC)n and (AAGAG)n repeats occupying the non-loop-forming regions. The *kl-3* loop is composed of a thinner filament and exhibits a rather diffuse appearance. Only (AATAT)n repeats have been mapped to the *kl-3* loop-forming region [1].

Y-loop generation reflects the high transcription levels of the underlying genes. Proper transcription of giant genes requires high processivity of RNA polymerase II (Pol II). The presence of long satellite arrays in the introns can lead to the slowing of elongation or frequent premature dissociations of RNA polymerase. Satellite repeats can form high-order DNA, RNA, or DNA–RNA hybrid structures, which may inhibit transcription elongation. Thus, transcription of gigantic intron-containing genes requires precise regulation, and Y-loops contain chromatin associated with a large number of transcripts and regulatory proteins [77,79]. Three RNA-binding factors, Blanks, Hephaestus (Heph), and Maca, are found to be enriched specifically in the chromatin of the Y-loops [74,80,81]. The corresponding mutations lead to male sterility owing to defects in sperm individualization, similar to the ones observed in males with knockdowns of the *kl-5*, *kl-3*, and *kl-2* genes [74,81]. These proteins are required for transcription or proper processing of the Y-loop gene transcripts. Blanks is found to be located to the Y-loop B (comprising mainly *kl-3* introns) and is needed for proper *kl-3* mRNA expression [74,80]. It has been assumed that Blanks maintains Pol II processivity by binding to the nascent transcripts of *kl-3* [74]. The Heph protein is found to colocalize with Y-loops A and C. It can regulate the expression of *kl-3*, *kl-5*, and *ks-1* mRNAs in spermatocytes. Heph may be involved in the processing of *kl-5* transcripts, including splicing, or in preventing their premature degradation [74]. Maca is essential for *kl-2* and *kl-3* transcription and proper splicing of *kl-3* transcripts, because in *maca* knockdown testes, the skipping of exon 13 causes an internal deletion in Kl-3 protein [81]. Recently described testis-enriched transcription regulators, tPlus3a and tPlus3b, appear to be required for the expression of fertility factors *kl-3* and *kl-5* [82] via an unknown mechanism. Some other RNA-binding proteins are enriched on the Y-loops in the spermatocyte nuclei, such as Pasilla and Boule [79,83], Rb97D [84], and several proteins encoded by unannotated genes (*lup-3*, *lup-4*, *dolly-1*, and *dolly-2*) [85]. However, their functions in Y-loop transcription await further investigation.

Y-loop generation is a conservative feature across the *Drosophila* genus, including *Drosophila simulans, Drosophila yakuba, Drosophila pseudoobscura, Drosophila littoralis*, and *Drosophila hydei* [86,87,88,89,90]. In the spermatocytes of *D. hydei,* clearly cytologically visible Y-loops are found, and early studies have uncovered that their transcription is associated with the huge DNA repeats [91,92,93]. Although the functional relevance of the gigantic introns still remains unclear, according to some assumptions long lasting transcription of fertility factor genes (around 80–90 h), due to the presence of the gigantic introns, appears to function as a ‘developmental timer’ for spermatocyte growth and differentiation [74,81]. Intron size could also play a critical role in the regulation of gene expression. It has been shown for the *Ultrabithorax (Ubx)* gene in the early *Drosophila* embryo that its large size causes abortive *Ubx* transcription during the syncytial divisions, blocking expression of Ubx protein at the syncytial stage [94].

### 3.2. Intron Gigantism in Humans

The phenomenon of ‘intron gigantism’ occurs across multiple species, including vertebrates, however, little data are available about its functional significance. Several human neuronal and muscle genes are known to bear giant introns. The best-known largest human gene is *dystrophin* comprising nearby 0.1% of the whole genome, containing 79 exons and spanning 2.2 Mb, with only 11 kb of coding sequence [95,96]. Its gigantic introns, also rich in repetitive DNA sequences, are reminiscent of those of Y-linked *Drosophila* fertility factors. *Dystrophin* is located on the p21 region of the X chromosome and codes the causative gene for Duchenne Muscular Dystrophy (DMD) and Becker Muscular Dystrophy (BMD) [95,97]. Dystrophin is a major scaffolding component of normal muscle, which links cytoskeletal actin, tubulin, and intermediate filaments to the extracellular matrix, and stabilizes the plasma membrane of striated muscle cells. Loss-of-function mutations of the *dystrophin* gene trigger instability of the plasma membrane and lead to myofiber loss [97,98]. Transcription of *dystrophin* takes place from several promoters in a tissue-specific manner. Full-length Dystrophin is expressed in all striated skeletal, smooth, and cardiac muscles. Shorter isoforms are expressed in brain and retina cells. In case of frameshift mutations, deficiency of the Dystrophin protein leads to severe DMD disease. In case of in-frame mutations, Dystrophin is expressed as a set of mutated proteins either with missense substitutions or deletions or duplications of its internal part, leading to the weaker BMD disease [97]. Exon skipping, with the aid of antisense oligonucleotides to skip the problem exons containing premature stop codon mutations or reading-frameshift mutations, is currently used as an approach for DMD therapy [99].

Transcription of these extremely large genes and the processing of their transcripts, including splicing, has a high metabolic cost for cells. The study of genes possessing giant introns using the *Drosophila* model provides a useful insight into the problems of expression of such genes in humans and the pathologies associated with their improper transcription or splicing. Study of the molecular mechanisms of maintenance, transcription, and processing of Y-loop genes in *Drosophila* may improve understanding of the origin, selection, and regulation of genes with similar structures in other species.

## 4. Differential Expression of rDNA Loci of *Drosophila* Sex Chromosomes

### 4.1. Nucleolar Dominance as a Widespread Phenomenon

In eukaryotes, there is a known phenomenon of a different level of expression of genes represented in the genome by two or more alleles [100,101]. Some of these alleles are expressed at a high level, while the expression of the rest of them is completely suppressed. One of the most striking examples of this phenomenon is the regulation of expression of loci encoding ribosomal RNA (rRNA), called nucleolar dominance. This phenomenon was initially discovered in interspecies hybrids of different taxonomic groups of animals [102,103,104]. In interspecies hybrids between *D. melanogaster* and *D. simulans*, rRNA genes from the *D. melanogaster* genome are predominantly expressed, while these genes from the *D. simulans* genome are suppressed [105]. However, later this process was also found within species. Nucleolar dominance has been observed in both the plant and animal kingdoms and is generally characterized by the dominant transcription of rRNA loci residing in only one chromosome [106,107,108]. Among the reasons for this phenomenon, DNA cytosine methylation, histone methylation and deacetylation, small RNA functions, and different affinities between transcription factors and promoter sequences of ribosomal DNA (rDNA) loci are suggested [109,110]. However, the exact mechanism of this phenomenon remains unclear to date.

rDNA loci are arranged as tandem repetitive rRNA gene clusters flanked by intergenic spacer sequences (IGSs) [109,110]. They are transcribed by the RNA polymerase I machinery as long precursor transcripts, subsequently processed into mature ribosomal RNAs (18S, 5.8S, and 28S). The transcriptional activity of these loci is high and achieves about 50–60% of the total transcription of metabolically active cells [102,111]. rRNAs are highly conserved, but the loci that encode them are among the most unstable elements of the genome due to their repetitive nature and high transcriptional activity. The number of copies of rRNA genes varies from 100 to 1000 in different organisms, and they are often distributed over many chromosomes, including ten loci in humans [112]. In mice, about 200 rDNA repeats grouped into NORs (nucleolar organizer regions) are distributed among the short arms of six acrocentric chromosomes [102]. rDNA can undergo intrachromatid recombination, which can lead to a loss of rDNA copies or to the formation of circular non-genomic units-extrachromosomal circular rDNAs (ERCs) accumulating in aging yeast cells [113]. In addition, active transcription of rDNA occurs even during the S-phase of the cell cycle, which can cause a conflict between replication and transcription. Such conflicts lead to frequent double-strand breaks and rDNA instability [114]. Given the multifactor nature of rDNA instability, the number of rDNA copies in the loci can vary greatly even within populations of the same species. For instance, in *D. melanogaster* strains, the variation in the number of rDNA copies can reach a sixfold range [115]. Similar differences in the number of rDNA copies have been shown for a number of other organisms, including mice and humans [116]. Decreased rDNA copy number leads to so-called replicative senescence in yeast [113,117,118]. Nevertheless, despite significant variations, there are mechanisms that maintain the number of rDNA copies both in populations and in the process of transmission to subsequent generations. Therefore, it is important to study the mechanisms of maintenance of rRNA gene copies at a level necessary for survival.

### 4.2. Y-Based Nucleolar Dominance in D. melanogaster Males

In *D. melanogaster*, rDNA loci reside on the X and Y chromosomes, each containing from 100 to 360 copies of rDNA genes. The presence of rDNA loci on the sex chromosomes greatly simplifies their study compared to other model organisms in which such loci are numerous and distributed over a large number of chromosomes. In *D. melanogaster* males, intraspecies epigenetic silencing of X chromosomal rDNA in males was shown by two research groups in 2012 [107,108]. However, these studies of nucleolar dominance were based on larval neuroblasts and total RNA preparations from adult flies. While rDNA gene transcripts on the X and Y chromosomes are highly homologous, some genes contain insertions of non-LTR retrotransposons *R1* and *R2* [119]. These retrotransposons are able to specifically recognize a 30 bp target sequence in the transcribed region of 28S rDNA and integrate into this region preventing correct transcription of the whole cistron. It has been suggested that the nucleolar dominance of Y-linked rDNA loci over those in the X chromosome is partly due to the different number of transposon insertions in the rDNA loci. X-chromosomal rDNA loci contain a higher proportion of genes disrupted by the transposons than Y-chromosomal ones [120]. For instance, the wild-type X chromosome contains insertions in 80% of rDNA units out of 100, while the wild-type Y chromosome contains insertions in 60% of rDNA units out of 360 [107]. The Y chromosome, with approximately the same number of insertions in the rDNA loci, does not show complete dominance over the X chromosome, but provides codominance (some expression of rDNA from the X chromosome occurs). Thus, there must be other factors that, together with the abundance of insertions, contribute to the nucleolar dominance of the Y chromosome. Interestingly, all analyzed lines of *Drosophila* whose Y chromosome did not exhibit complete dominance carried mutations in the genes of heterochromatin proteins *Su(var)2–5* (HP1) and *Su(var)3–9* (encoding histone H3K9 methyltransferase). However, the authors failed to establish an unambiguous relationship between mutations of heterochromatin protein genes and derepression of rDNA loci in the X chromosome [107]. Previously, it has been shown that in the absence of H3K9 methylation (in the case of a *Su(var)3–9* mutation) and upon disruption of the siRNA pathway (a *dcr-2* mutation) disorganization of nucleoli, rDNA loci, and adjacent satellite DNAs were observed [121]. Recently, nucleolar dominance was analyzed in detail in *dcr-2* and *Su(var)3–9* mutants. Males with the *dcr-2* mutation showed no significant change in Y chromosome dominance during fly development, while males with the *Su(var)3-9* mutation demonstrated a significant decrease in Y dominance in nervous tissue of larvae, imaginal discs, and germline stem cells (GSCs) of adult males [122]. Thus, heterochromatin-mediated repression of rDNA loci may contribute to the mechanism that regulates of their activity.

A recent work describes the investigation of nucleolar dominance of Y-linked rDNA loci in male GSCs of *D. melanogaster* [123]. Although in the testes of young males, most GSCs contain a single spherical nucleolus 2 µm in diameter; however, during aging, the proportion of GSCs with normal nucleolus morphology gradually decreased, while the proportion of GSCs with atypical morphology increased. Atypical nucleolus morphology was manifested both in the fragmentation of the nucleoli into several foci, or in the altered nucleolar shape. Authors found that only Y-linked rDNA loci are associated with the nucleolus with typical nucleolar morphology, while X-linked loci are not, regardless of age. These results suggest that Y-chromosomal rDNA is actively transcribed, while X rDNA is not, which is consistent with Y nucleolar dominance. However, atypical nucleolar morphology that occurs in GSCs with aging is associated with the activation of the silent rDNA loci on the X, and leads to the transcription of rDNA from two separate chromosomes, each of which forms a separate nucleolus. Activation of X rDNA probably compensates for the decrease in the number of active copies of Y-linked rDNA, which decreases during aging owing to conflicts between transcription and replication machineries causing rDNA instability [114,123]. GSC nucleolar morphology and rDNA copy number reduction is heritable and passed to male offspring from old fathers. Strikingly, the authors find that nucleolar morphology can be recovered in individual GSCs of these F1 sons during the first 10 days after eclosion to restore normal Y dominant state of rDNA transcription. This indicates the existence of a mechanism to maintain the number of rDNA copies across generations. This mechanism may be adaptive for the following reasons: firstly, rRNA expression from only one chromosome can prevent rDNA deletions on the other chromosome, transcription from which is suppressed; secondly, having intact rDNA loci present may allow GSCs to prolong their lifespan [123].

Recently, the SNP in situ hybridization method was used to analyze in detail the transcription of rDNA clusters from the X and Y chromosomes of *D. melanogaster* [122]. Throughout *Drosophila* male development, the codominance of X and Y rDNA loci changes to the dominance of those on the Y chromosome. The manifestation of Y dominance in most types of larval tissues, such as nervous tissue, imaginal discs, fat body, and enterocytes of the anterior part of the midgut, has been found. However, salivary glands containing a large number of polytenized chromosomes showed only a modest manifestation of Y dominance. In females, using the SNP method, the codominance between the two X chromosomes was confirmed. *Drosophila* females with the XXY genotype also exhibit Y dominance, suggesting that the presence of the Y chromosome is necessary and sufficient for the dominance. However, in the ovaries of adult females, codominance is also observed in GSCs and cystoblasts, while in the nurse cells Y dominance is found. Thus, in the case of the presence of the Y chromosome, nucleolar dominance predominantly occurs independently of the sex of the cell. This leads to the assumption that the Y chromosome must contain a specific nucleotide sequence that allows dominance to occur. In general, the sequences in rDNA loci of the Y chromosome and/or its proximal regions may be essential for the nucleolar dominance. Moreover, these results do not exclude the possibility that the long arm of the Y chromosome is involved in this process [122].

### 4.3. Non-Random Segregation of Sister Chromatids of Sex Chromosomes in Drosophila

Recent studies also point to the involvement of rDNA loci in the nonrandom segregation of sister chromatids during cell division. The intergenic spacer repeats are responsible for X-Y pairing in *D. melanogaster* males [124]. Sister chromatids are not always completely identical due to the presence of epigenetic marks that distinguish them. The asymmetric arrangement of these marks, as well as kinetochore proteins, can lead to selective recognition of chromatids. The divergence of such sister chromatids is apparently one of the causes of asymmetric cell division. Recent studies have shown that non-random sister chromatid segregation is mediated by rDNA loci [125]. Earlier it was shown that non-random segregation in *Drosophila* is characteristic of the sister chromatids of the X and Y chromosomes, but not the autosomes [126]. Researchers used the *Drosophila* strain carrying a deletion of 80% of heterochromatin in the wild-type X chromosome (*Df(1)bb^158^*). In GSCs of such fly males, random segregation of X chromosome chromatids occurred, while the segregation of the Y chromosomes remained non-random, suggesting that a chromosomal element deleted in the *bb^158^* strain acts *in cis* to mediate non-random sister chromatid segregation. This indicates that the genetic elements necessary for this phenomenon are present in the deleted heterochromatin. It can be concluded that this genetic element is located in the rDNA loci. A more detailed study revealed that IGS sequences and the protein that binds to these sequences, Indra, are responsible for the non-random segregation of sister chromatids [125]. Unequal sister chromatid exchange can be proposed as a possible mechanism to increase rDNA copy number on one sister chromatid for restoration of the number of rDNA copies disrupted in GSCs by aging.

### 4.4. Differential Expression of rDNA Loci in Human

The phenomenon of nucleolar dominance appears to be common across multiple species. It has not been shown directly in humans, due to the distribution of rDNA loci in multiple autosomal regions making them difficult to analyze owing to their highly repetitive nature. However, only a part of rDNA loci is actively transcribed in human cell lines [127,128,129], suggesting that these loci may also undergo activation or suppression. To date, the principles of silencing or activation of rDNA loci in humans remain unknown. Recently, with the aid of Oxford Nanopore sequencing technology, obvious differences between methylated and unmethylated rDNA gene arrays in human cells have been revealed. The ratio of transcriptionally active unmethylated copies versus methylated ones has been found to be lower in individuals with higher rDNA copy abundance, indicating a possible mechanism for maintenance of a stable number of active rDNA copies [129].

## 5. *Drosophila* Y Chromosome in Studying of piRNA Biogenesis and Functioning of piRNA-Clusters

### 5.1. Brief Description of the piRNA System

**Figure 3:**
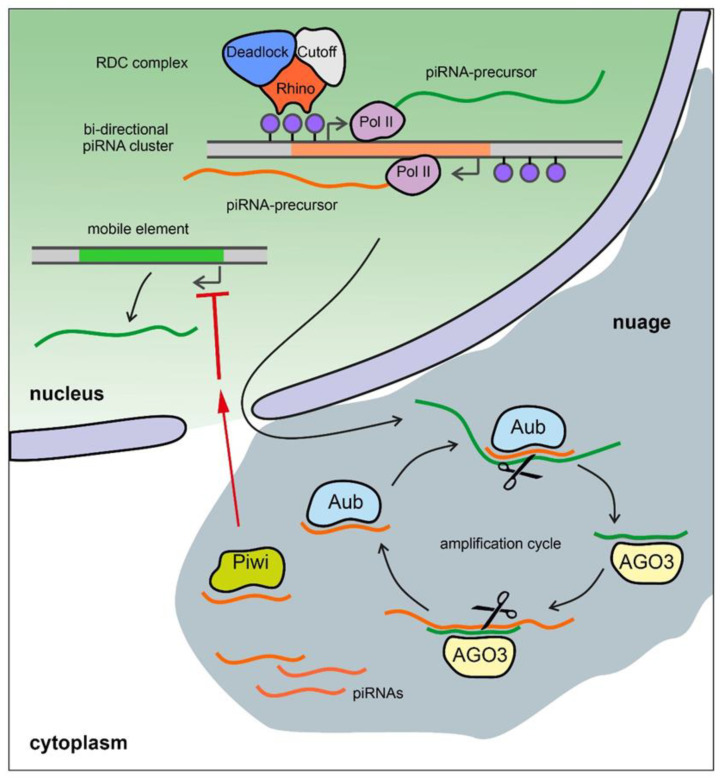
piRNA biogenesis in *Drosophila* germ cells. Bi-directional piRNA clusters are recognized by the RDC complex with the aid of histone modification H3K9me3 (blue dots) and are transcribed by Pol II machinery with the production of long unspliced transcripts of piRNA precursors. They are exported from the nucleus in the perinuclear nuage granules and are presumably cleaved by endonuclease Zucchini forming the 5′-end of the future piRNA (not shown). The cleaved transcripts are loaded into PIWI clade protein Aubergine (Aub) and then trimmed from the 3′-end by an unknown trimmer nuclease (not shown). Aub loaded with guide antisense piRNA recognizes and cleaves the complementary sense transcript producing the 5′-end of a new sense piRNA. The new piRNA is loaded into PIWI clade protein AGO3 and, in turn, performs cleavage of the complementary antisense transcript. This step generates a new antisense piRNA that is identical or very similar to the initiating piRNA (ping-pong amplification cycle). Piwi proteins loaded by antisense piRNAs translocate into the nucleus where they suppress transcription of TEs with complementary sequences by a co-transcriptional repression mechanism.

The piRNA pathway provides both innate and adaptive immune system defense against the activity of transposable elements (TEs) leading to the protection of genome integrity in germinal tissues. It also participates in the maintenance of germline stem cells, regulation of protein-coding gene expression, the establishment of embryonic patterning (in Diptera), and transgenerational epigenetic inheritance [130,131,132]. Small non-coding piRNAs 23-35 nt in length associated with proteins of the PIWI subfamily are present in animals from fungi to humans [133,134,135]. piRNAs are generated from piRNA clusters, which are long precursors that are transcribed from heterochromatic regions containing fragments of transposons. piRNA precursors are processed to generate small piRNAs in perinuclear nuage granules. Mature piRNAs loaded into the proteins of the PIWI subfamily, forming piRNA-induced RNA silencing complexes (piRISCs). The generation of primary piRNAs triggers production of secondary piRNAs via an amplification system called the ping-pong cycle (Figure 3) [136]. The piRNA pathway is active, as a rule, in gonads and plays an essential role in fertility maintenance, preventing transposon activity and repressing harmful protein-coding genes. Transcripts of harmful genomic elements can be silenced post-transcriptionally via recognition and cleavage of complementary RNA-targets by piRISC complexes in the nuage. There is also a co-transcriptional repression mechanism, where recognition of nascent transcripts by piRISCs loaded with guide piRNAs leads to the establishment of heterochromatin in the corresponding genomic regions. Most of the known piRNA clusters in *Drosophila* are bidirectional and transcribed with the participation of a specific Rhino–Deadlock–Cutoff (RDC) complex (Figure 3) [137,138,139]. Due to the Rhino chromodomain the RDC complex recognizes H3K9me3 histone modifications enriched in the chromatin of piRNA clusters and recruits to them the transcription initiation factor Moonshiner to promote non-canonical transcription. The RDC complex allows a skipping of transcription termination sites and inhibits splicing of piRNA precursor transcripts [137,138,139]. In contrast, in mammals, the mechanisms of piRNA cluster expression seem to be indistinguishable from canonical Pol II transcription and include regular splicing and polyadenylation of the transcripts [131]. However, the role of piRNAs may not be limited to germinal tissues. The involvement of the piRNA pathway in processes associated with disease, such as tumors of various etiologies, and aging in humans, has recently been shown [140,141].

### 5.2. The Y Chromosome as a Major piRNA-Producing Genomic Region in the Fly Testes

The piRNA system in *D. melanogaster* exhibits a strong sexual dimorphism. TE-mapping piRNAs are known as the most abundant class of piRNAs in the ovaries, whereas only about 40% of piRNAs map to TEs in the testes, and the largest cohort of piRNAs map to protein-coding genes [7,142]. The genomic origin of most piRNAs between the two sexes is also different. In the testes of *Drosophila*, almost half of all piRNAs originate from the piRNA clusters located on the Y chromosome (Figure 1a) [7]. The largest number of piRNAs is generated from the Y-linked *Suppressor of Stellate* (*Su(Ste)*) repeats directed to silencing of the homologous tandem *Stellate* genes residing on the X chromosome [142,143,144]. The organization of the *Su(Ste)* loci has been studied in detail. The number of *Su(Ste)* repeats comprises more than 500 tandemly ordered copies residing in two cytolocations on the Y (Figure 1a) [2,4,7]. The size of a typical *Su(Ste)* repeat is about 28 000 nt, consisting of three main parts: region homologous to the *Stellate* genes, the AT-rich Y-specific region, and the insertion of transposon *hoppel* (*1360*) into the promoter. *Su(Ste)* repeats are transcribed and processed to polyadenylated mRNAs; however, they contain numerous frameshift mutations owing to the presence of point mutations and deletions, and they are not translated [145]. The insertion of the defective transposon *hoppel* is responsible for the initiation of antisense transcription of *Su(Ste)* repeats and their acquisition of piRNA cluster functions [143]. *Stellate* derepression in the case of deletion of most of *Su(Ste)* repeats or disruption of the piRNA system leads to the accumulation of needle-like protein aggregates in spermatocytes, disturbances of meiosis, and, as a result, a decrease in male fertility [143,146]. The *Stellate/Su(Ste)* system is species- and sex-specific for *D. melanogaster*. Earlier, it was proposed that *Stellates* are selfish genes involved in meiotic drive [147]; however, no experimental pieces of evidence of this assumption have been found to date. Recently, it was is shown that *Stellate* genes participate in male hybrid sterility of F1 progeny of crosses between *D. melanogaster* females and *D. mauritiana* males. The hybrid males possess maternal X-linked *Stellate* genes, but their paternal Y chromosome does not contain *Su(Ste)* repeats and the corresponding piRNAs are not generated. Derepression of *Stellates* in the testes of hybrid males leads to a meiotic catastrophe and complete sterility [142,146]. The contribution of the *Stellate/Su(Ste)* system to reproductive isolation may explain the fixation and maintenance of this system in the *D. melanogaster* genome. The acquisition of the *Stellate/Su(Ste)* system by a part of the ancient fruit fly population could have been a causative factor of hybrid sterility in crosses of females with males that do not possess *Su(Ste)* repeats on the Y. According to another speculation, the *Stellate/Su(Ste)* system is similar to toxin–antitoxin systems, which are widespread in prokaryotes [148].

Considering the amplification processes as an inherent property of the Y chromosomes, it can be assumed that some amplified repeats can be licensed as piRNA clusters during the evolution of a species. Given that not all piRNAs map to TEs, piRNAs produced by regions of the Y could exert sex-specific functions to regulate the expression of protein-coding genes besides *Stellate*. If a gene has a positive effect on the processes occurring in the ovaries or other tissues, but, at the same time, reduces the efficiency of spermatogenesis, mechanisms for its testis-specific suppression can be developed, including piRNA-mediated silencing. The existence of a similar mechanism has been recently confirmed for the X-chromosomal *pirate*/CG12717 gene, encoding a SUMO-isopeptidase. The Y chromosome of *D. melanogaster* contains the *petrel* locus (Figure 1a), which is a source of multiple piRNAs highly complementary to *pirate*, providing strong testis-specific silencing of this gene [7]. However, the functional significance of the repression of *pirate* in the testes remains unclear to date. It appears that both in the cases of the *Stellate/Su(Ste)* and *pirate/petrel* pairs, their current evolutionary relationships are initially based on parallel acquisition or co-amplification of homologous genes on the sex chromosomes. Note that in the *Drosophila* testes, the expression and activity of the RDC complex is mainly limited to early stages of spermatogenesis, including GSCs and spermatogonial cells [149]. The transcription of Y-linked *Su(Ste)* and *petrel* piRNA clusters takes place in primary spermatocytes, and it is independent from the RDC complex [149]. The complex mosaic structure of *petrel* repeats [7] makes their further study as a functional piRNA cluster difficult. In the case of *Su(Ste)*, its sense transcription performs in the canonical manner from its own promoter, and the antisense transcription is initiated from several sites within the inserted transposon *hoppel* [143,150], which makes it similar to mammalian piRNA clusters.

It has been assumed that in *Drosophila*, maternal piRNAs, which are stored at the posterior pole of the oocyte during oogenesis, ensure the initiation of piRNA biogenesis from long piRNA precursors. However, this does not apply to Y chromosomal piRNA clusters due to the absence of the Y chromosome itself in females. According to a recent study, the determination of long RNAs as primary piRNA sources can also occur due to the recognition of specific *cis*-regulatory 100-nt elements in piRNA precursor sequences, as in the case of the long non-coding RNA *flamenco* and 3′ UTR of *tj* mRNA in ovarian somatic cells [151]. In many animals, including humans, the induction of germ cell precursors occurs from somatic pluripotent epiblast cells during embryogenesis, as a result of which all piRNA clusters are determined de novo [132,135]. On the whole, the mechanism of determination of genomic regions as piRNA clusters is poorly resolved. Future studies of Y-chromosomal piRNA clusters in *Drosophila* could allow us to elucidate these mechanisms both in *Drosophila* and in mammals.

### 5.3. The Y Chromosome in Other Species as a Source of piRNAs

The suppression of genes harmful for spermatogenesis appears to be one of the main functions of piRNAs originating from the Y chromosome of *D. melanogaster*. In mouse testes, novel polyadenylated non-coding RNAs called *Pirmy* and *Pirmy*-like transcribed from the long arm of the Y chromosome have recently been discovered [152]. Multiple splice variants of *Pirmy* encoded by a single locus have been identified experimentally; however, each exon of *Pirmy* has been also found to be amplified in multiple copies on the Y chromosome. The 28 *Pirmy*-like RNA variants present various combinations of these exons that are distributed in multiple different loci on the mouse Y chromosome. Morphology- and sperm motility-related abnormalities have been found in two strains of Y-deleted mice with disrupted expression of *Pirmy* and *Pirmy*-like RNAs. The *Pirmy* and *Pirmy-like* RNAs serve as sources of piRNAs that are complementary to 5′- and 3′-UTRs of several autosomal genes, such as *FABP9*, *Spink2*, *superoxide dismutase (SOD)*, and *calreticulin*, and also genes that presumably contribute to sex ratio maintenance in the progeny. The proteins expressed from these autosomal genes are up-regulated in the sperm of Y-deleted mice and appear to be responsible for the disruption of sperm morphology and motility [152].

In *Bombyx mori*, females are the heterogametic sex (ZW), and the W chromosome is heterochromatinized and consists almost entirely of transposon sequences. piRNA from the *Fem* locus on the W chromosome functions as a suppressor of the *Masc* gene, which regulates sex-specific splicing of the *doublesex (dsx)* gene, which is necessary for sex determination in many insects [153]. Thus, small piRNAs from the Y or W chromosome can potentially be involved in sex determination, the resolution of intragenomic conflicts, reproductive isolation, and the regulation of gene expression for ensuring spermatogenesis [7,16,142,151,152,153,154].

Y-linked piRNA clusters and their functions in humans remain poorly understood [155,156]. High-throughput sequencing of piRNAs from three human adult testis samples and subsequent data analysis have revealed 28 putative piRNA-cluster candidate regions on the Y [157]. However, among them, only one uni-directional cluster with coordinates chrY:3231747-3235845 contains a significant number of mapped piRNAs (45.4 rpkm). This locus includes remnants of SINE, LINE, and LTR TEs, and has a highly homologous region of the same size on the X chromosome. The remaining 27 piRNA clusters predicted on the Y are rather small and remain unexplored. It should also be noted that due to the high level of heterochromatinization and a large number of repetitive elements, the human Y chromosome is not perfectly assembled, and data about piRNA clusters are not complete.

### 5.4. The Y Chromosome and TEs

Degeneration of the Y chromosome has been accompanied by the acquisition of transposable elements. The old Y chromosome of *D. melanogaster* is strongly enriched with retrotransposons of different families. Earlier, in some laboratory strains of *D. melanogaster,* active Gypsy elements restricted to the Y chromosome have been found [158]. Using the latest genome assembly, it has been uncovered that *Dm412*, *Gypsy*, *Het-A*, *Doc*, *TART*, *Mdg1*, *Mdg3*, *blood*, and *FW* TEs are prevalent on the Y chromosome of *D. melanogaster* [4]. Y chromosomes of the *D. simulans* clade are similarly enriched in retrotransposons relative to the rest part of the genomes; however, Y chromosomes from even closely related species accumulate distinct TE sets [5]. Whether TE accumulation is coupled with beneficial developmental processes remains to be determined. The stability and non-random localization of TEs throughout the Y speaks in favor of their putative functional role in the host [2,159]. Recently the organization of functional centromeres of *D. melanogaster* has been resolved in detail, due to the mapping of CENP-A-occupied regions of all chromosomes. It has been found that CENP-A mapped DNA is mainly composed of retrotransposons and is often flanked and inserted by large blocks of satellite repeats [160]. However, satellites are practically not found in the Y centromere, despite the fact that the whole Y chromosome is strongly enriched in simple tandem repeats [4]. For instance, the Y centromere region consists of a tandem array of non-LTR mobile element *Jockey-3* [160], and its stability and active state is required for the maintenance of the centromere. The presence of telomere-specific *Het-A*, *TAHRE*, and *TART* non-LTR retrotransposons in the pericentromeric region of the *D. melanogaster* Y chromosome and in closely related species [2,161] remains mysterious and can potentially be associated with their involvement in telomere maintenance [162].

Recent data indicate that the Y chromosome of *D. melanogaster* appears to be a cryptic library of active copies of TEs. The preference of TEs for insertion into the Y chromosome can potentially be beneficial for the host, providing immunity against active TEs in the testes by producing piRNAs. However, by analyzing testis single-cell sequencing data, Lawlor and colleagues found an unexpected burst of activity of TEs residing on the Y in early spermatocytes of *D. melanogaster* [163]. This event occurs during a specific developmental period that coincides with the up-regulation of Y-chromosomal fertility factors and spermatocyte-specific transcription of many Y-linked genes [73], as well as with a decreasing level of several components of the piRNA pathway. Piwi expression is not detected in primary spermatocytes [164,165], nor is the RDC complex [149]. As mentioned above, the RDC complex is responsible for transcription of bi-directional piRNA clusters providing the bulk of piRNAs for the suppression of TEs. Indeed, in primary spermatocytes, the piRNA machinery switches to the production of piRNAs from the non-canonical *Su(Ste)* and *petrel* clusters, ensuring the silencing of protein-coding genes [7,142,143]. Note that moderate activation of TEs at this stage of spermatogenesis can, in a certain percentage of cases, lead to transposon insertions and mutations in functional genomic regions. Given that spermatogenesis is a highly redundant process, some TE activity leading to detrimental effects with a low frequency may be inconsequential. Eventually, some mutations can be adaptive for an individual and, will subsequently be fixed in the population. Thus, TE mobilization in this narrow developmental window leads to newly arising genetic variability important for the evolutionary adaptation of the population to changing environmental conditions.

## 6. Conclusions

Despite the fact that in some animal taxa Y chromosomes are completely absent, in most heterosexual eukaryotes Y chromosomes are maintained and perform various essential functions. These include sex determination, ensuring male fertility; correct segregation of meiotic chromosomes; regulation of the activity of rDNA repeats; epigenetic regulation of harmful elements, including TEs and protein-coding genes; contribution to interspecies hybrid sterility, and other responsibilities. The Y chromosome life cycle progresses through a series of stages common to many organisms, such as birth, accumulation of genes necessary for spermatogenesis, cessation of the recombination process, degeneration of the bulk of acquired sequences, and aging. Whereas in mammals the appearance of the Y chromosome has occurred once in a common ancestor of marsupials and placentals before their splitting, presumably 160-180 MYA, in Diptera and some fishes, Y chromosomes have arisen and disappeared several times during their evolutionary history. Studies of model organisms, *Drosophila* and mice, have fundamental significance for uncovering the shared properties of Y chromosomes of multiple species. The convergent nature of evolution of the Y chromosome allows researchers to consider that the data obtained in model organisms can be useful to a certain extent for the prediction of the human Y chromosome behavior in the future, as well as in understanding how the specific structure of this chromosome reflects its functions in normal and pathological conditions.

## Data Availability

Not applicable.
